# Removal of the Uterus with Double Ovariotomy

**Published:** 1885-07

**Authors:** 


### Removal of the Uterus with Double Ovariotomy.

Dr. Gorde, in the *British Medical Journal*, reports a case of double ovariotomy in which the uterus was also removed. The patient was admitted into Prince Alfred Hospital, Sydney, New South Wales, with marked ovarian disease. She was a widow, aged sixty, and had had twelve children, the youngest being seventeen years old. She had menstruated irregularly for several years—sometimes profusely—when six years before it ceased altogether. The operation was performed under the influence of methylene-bichloride. Both ovaries were found diseased, the left being the size of a large walnut; the right had become a large unilocular cyst, firmly adherent to the uterus, but otherwise there were no adhesions. The cyst contained thirteen pints of a thin greenish fluid of specific gravity 1.038. This having been removed, the adhesions to the uterus were found so firm that it was deemed advisable not to attempt to separate them, but rather to remove the uterus also, as by so doing the peritoneum would be less disturbed and the chances of the woman's recovery possibly increased. He accordingly passed, with an aneurism needle, a double ligature of strong cable-laid carbolized silk, one-half of which was tied round the lower part of the cervix uteri, and the other half included the remaining attachments. The cervix was then cut through, the other ovary removed and the pedicle returned. The patient made a rapid recovery, the temperature never rising above 101.4° F. On the thirteenth day she was allowed to get up and lie upon a couch. On the fifty-ninth day she left the hospital feeling perfectly well. The chief point of interest about the case is the fact that the pedicle, which consisted of the lower third of the cervix uteri, was secured with a single silk ligature, and returned into the abdominal cavity with the mucous membrane of the cervical canal entire.

